# Calcium enhanced the resistance against *Phoma arachidicola* by improving cell membrane stability and regulating reactive oxygen species metabolism in peanut

**DOI:** 10.1186/s12870-024-05222-1

**Published:** 2024-06-05

**Authors:** Lanshuo Yan, Sichao Liu, Ruoxin Li, Zibo Li, Jingzi Piao, Rujun Zhou

**Affiliations:** Department of Plant Pathology, College of Plant Protection, Shenyang Agricultural University, Shenyang, Liaoning China

**Keywords:** Peanut, Web blotch disease, Cell membrane permeability, Reactive oxygen species, Antioxidant enzymes

## Abstract

**Background:**

Peanut (*Arachis hypogaea*), a vital oil and food crop globally, is susceptible to web blotch which is a significant foliar disease caused by *Phoma arachidicola* Marasas Pauer&Boerema leading to substantial yield losses in peanut production. Calcium treatment has been found to enhance plant resistance against pathogens.

**Results:**

This study investigates the impact of exogenous calcium on peanut resistance to web blotch and explores its mechanisms. Greenhouse experiments revealed that exogenous calcium treatment effectively enhanced resistance to peanut web blotch. Specifically, amino acid calcium and sugar alcohol calcium solutions demonstrated the best induced resistance effects, achieving reduction rates of 61.54% and 60% in Baisha1016, and 53.94% and 50% in Luhua11, respectively. All exogenous calcium treatments reduced malondialdehyde (MDA) and relative electrical conductivity (REC) levels in peanut leaves, mitigating pathogen-induced cell membrane damage. Exogenous calcium supplementation led to elevated hydrogen peroxide (H_2_O_2_) content and superoxide anion (O_2_^∙-^) production in peanut leaves, facilitating the accumulation of reactive oxygen species (ROS) crucial for plant defense responses. Amino acid calcium and sugar alcohol calcium treatments significantly boosted activities of peroxidase (POD), superoxide dismutase (SOD), catalase (CAT), and ascorbate peroxidase (APX) in peanut leaves. Activation of these antioxidant enzymes effectively scavenged excess ROS, maintaining ROS balance and mitigating cellular damage.

**Conclusions:**

In summary, exogenous calcium treatment triggered ROS production, which was subsequently eliminated by the activation of antioxidant enzymes, thereby reducing cell membrane damage and inducing defense responses against peanut web blotch.

## Background

Peanut (*Arachis hypogaea*), rich in nutrients such as proteins, vitamins, minerals, polyphenols, and flavonoids, is a crucial source of oil originating from the tropical regions of South America [1, 2]. As the world’s leading producer, China has dedicated approximately 4684 thousand hectares to peanut cultivation, yielding around 18.329 million tons in 2022 (National Bureau of Statistics data, http://www.stats.gov.cn/). However, various biotic and abiotic stresses affect peanut yield, with pests and diseases being among the major factors causing yield reduction [3]. Peanut is susceptible to a multitude of pathogens, with common diseases including peanut black spot, peanut early leaf spot, and peanut web blotch, the latter being the most severe and caused by *Phoma arachidicola* Marasas Pauer&Boerema [4]. Peanut web blotch primarily occurs during the mid to late stages of peanut growth, especially during the pod-setting and maturing phases, leading to significant defoliation and yield losses. It can generally result in a 10–20% reduction in peanut yield, with losses exceeding 30% in severe cases, thereby posing a serious threat to global peanut production [5]. At present, the predominant strategy for managing peanut web blotch in agricultural production involves the application of chemical fungicides. However, the long-term and frequent application of fungicides can lead to a series of severe problems, including pathogen resistance, excessive pesticide residues in agricultural products, and environmental pollution, thereby degrading peanut quality, constraining sustainable agricultural development, and affecting human health [6–8]. Therefore, finding practical and effective control methods that are harmless to the environment and human health has become a hot topic in peanut disease management.

Compared with traditional chemical fungicides, the use of plant immune elicitors is a more environmentally friendly and sustainable plant protection strategy. It enhances the plant’s resistance to pathogens and other stress factors by stimulating the plant’s own defense system, thereby increasing crop yield and economic benefits [9, 10]. Several studies have confirmed that many synthetic and natural chemicals can act as elicitors, such as calcium chloride (CaCl_2_) [11, 12], benzothiadiazole (BTH) [13, 14], salicylic acid (SA) [15, 16], methyl jasmonate (MEJA) [17, 18], β-aminobutyric acid (BABA) [19, 20], chitosan [21, 22], and silicates [23, 24]. Within this context, calcium (Ca) is recognized as a pivotal element essential for plant growth, serving as a secondary messenger in plant signaling pathways. The application of various calcium compounds is acknowledged for its efficacy in augmenting plant resistance to both biotic and abiotic stresses [25, 26].

The role of calcium in inducing disease resistance operates through enhancing plant cell membrane stability, regulating reactive oxygen species metabolism, and activating antioxidant enzyme systems. Numerous studies have demonstrated that the application of exogenous calcium significantly enhances the levels of both free and bound calcium within plants [27]. This process not only preserves the integrity of cell walls but also maintains the stability of biological membranes. For instance, immersing post-harvest pears in calcium chloride has been shown to increase the formation of calcium phosphate and calcium oxalate on the fruit peel. Such treatments help to maintain the normal structure of cell wall pectin, consequently diminishing the ability of pathogens to invade and reproduce [28–30]. Research by Jiang et al. indicates that exogenous calcium application increases the activity of peroxidase enzymes and ROS levels in tomato plants, reducing the incidence of tomato bacterial wilt, suggesting that calcium can regulate the balance of the reactive oxygen species metabolism system, affecting the generation and scavenging of free radicals within cells, thus regulating the biomembrane system and metabolic processes to enhance plant disease resistance [31, 32]. Studies on soybeans by Sugimoto et al. have revealed that spraying calcium formate and calcium nitrate boosts defensive enzyme activities, significantly inhibiting the occurrence of soybean stem rot, indicating that calcium, as an important signaling molecule, can improve plant disease resistance by regulating specific antioxidant enzymes, such as superoxide dismutase, catalase, peroxidase, and ascorbate peroxidase [33, 34]. Furthermore, exogenous calcium also plays a very important role in enhancing the stress resistance and improving the quality of peanuts [35–37]. However, compared to vegetables and crops, the mechanism of calcium-induced resistance in peanuts is still not clear.

Calcium, as an elicitor for plant disease control, has potential applications, but its mechanism of inducing disease resistance is still unclear. To explore the potential application of calcium in resisting peanut web blotch and to further elucidate its role in the mechanisms underlying disease resistance in peanuts, this research was structured around four main objectives: (1) the inductive effect of exogenous calcium treatment on peanut web blotch; (2) the impact of exogenous calcium treatment on the permeability of peanut leaf cell membranes; (3) the effect of exogenous calcium treatment on the hydrogen peroxide content and superoxide anion production rate in peanut leaves; (4) the impact of exogenous calcium treatment on the activity of antioxidant enzymes in peanut leaves. The research findings will provide a theoretical basis for further understanding the application of calcium in the mechanism of peanut resistance to web blotch.

## Methods

### Peanut plants and pathogen preparation

The susceptible peanut variety Baisha1016 and the resistant variety Luhua11 (Dongya Seed) were selected as the study subjects [38]. The peanut web blotch pathogen was provided by the Plant Pathology Laboratory in the Plant Protection College of Shenyang Agricultural University. Spore propagation of the pathogen was conducted before inoculation, by cultivating on oatmeal agar (OA) medium under near-ultraviolet light at 23 ± 1 °C in an incubator for a duration of 14 days. The concentration of spore suspension was measured using a hemocytometer. The spore concentration for inoculation was adjusted to 1.0 × 10^5^ spores/mL using sterile distilled water. The whole peanut plants were inoculated by employing an artificial spray inoculation method.

### Exogenous calcium treatment and inoculation

Five calcium compounds (2000 mg/L calcium chloride, 2000 mg/L calcium nitrate, 1500-fold dilution of sorbitol calcium, 1000-fold dilution of amino acid calcium, and 1000-fold dilution of EDTA-Ca solution) were used for treatment [39]. 28 days after sowing, the peanuts were divided into 7 groups. Groups 1 and 2 were sprayed with clean water on the leaves, while groups 3, 4, 5, 6, and 7 were sprayed respectively with calcium chloride, calcium nitrate, sorbitol calcium, amino acid calcium, and EDTA-Ca solution. 48 h after calcium treatment, the prepared spore suspension was sprayed on the leaves of groups 2, 3, 4, 5, 6, and 7 for inoculation. Post-inoculation, the peanut plants within all groups were enclosed in humidity bags for 48 h to ensure consistent environmental conditions, culminating in a total of 7 distinct treatment groups. Each treatment was replicated three times, and samples were taken at 0, 12, 24, 48, 72, and 96 h post-inoculation. Peanut leaves from the same position were cut and immediately frozen in liquid nitrogen and stored at -80 °C for further analysis.

### Disease index and induced resistance effect

The development of the disease condition was monitored on the 7th, 14th, and 21st days after inoculation with the peanut web blotch pathogen. The severity grading standard for peanut web blotch disease was used for scoring: 0 level: no disease spots; 1 level: disease spot area covering less than 25% of the leaf area; 2 level: disease spot area covering 26%~50% of the leaf area; 3 level: disease spot area covering more than 50% of the leaf area; 4 level: severe leaf drop or plant death [40]. The disease index and induced resistance effect were calculated using Formula 1 and Formula 2, respectively.

1 $$\eqalign{& {\rm{Disease}}\,{\rm{index}}\,{\rm{(PDI)}}\,{\rm{ = }} \cr & {{{\rm{Sum}}\,{\rm{of}}\,{\rm{all}}\,{\rm{rating \times 100}}} \over {{\rm{Total}}\,{\rm{number}}\,{\rm{of}}\,{\rm{plants \times maximum}}\,{\rm{rating}}\,{\rm{grade}}}} \cr}$$

2 $$\eqalign{& {\rm{Induction}}\,{\rm{effect}}\,{\rm{(\% )}}\,{\rm{ = }} \cr & {\eqalign{& {\rm{Disease}}\,{\rm{index}}\,{\rm{in}}\,{\rm{control}}\,{\rm{plants}} \cr & {\rm{ - disease}}\,{\rm{index}}\,{\rm{in}}\,{\rm{treated}}\,{\rm{plants}} \cr} \over {{\rm{Disease}}\,{\rm{index}}\,{\rm{in}}\,{\rm{control}}\,{\rm{plants}}}} \times 100 \cr}$$

### Malondialdehyde (MDA) content and relative electrical conductivity (REC) measurement

The Malondialdehyde (MDA) content was determined using a commercial MDA content detection kit (Solarbio). To evaluate membrane leakage, the relative electrical conductivity (REC) was measured according to Dionisio-Sese and Tobita [41]. 0.1 g of peanut leaves from different treatments were rinsed with deionized water and dried with clean filter paper, then soaked in 10 mL room temperature deionized water for 12 h to measure their conductivity (R_1_). The test tubes were then heated in a boiling water bath for 30 min, cooled to room temperature, and shaken well before measuring the conductivity post-boiling (R_2_). The relative electrical conductivity was calculated using Formula 3.

3 $${\rm{REC}}\,(\% ) = {{R1} \over {R2}} \times 100$$

### Hydrogen peroxide (H_2_O_2_) content determination

The content of Hydrogen Peroxide (H_2_O_2_) was determined according to the method of Rao MV et al. [42]. 1 g of peanut leaves from different treatments were ground into powder in liquid nitrogen, placed in a centrifuge tube with 2 mL of precooled acetone, and mixed well into a slurry. The tubes were then centrifuged at 10,000 g at 4 °C for 10 min. 1 mL of supernatant was taken, mixed with 3 mL of extracting agent (CCl_4_-CHCl_3_, 3:1 by volume) and 5 mL of distilled water, and mixed well. After centrifugation at 5000 g for 1 min, the upper aqueous phase was used for H_2_O_2_ measurement. 1 mL of the test liquid was added to 2 mL of working solution (Reagent A: 3.3 mmolL^-1^ ferrous sulfate, 3.3 mmolL^-1^ ammonium sulfate, 412.5 mmolL^-1^ sulfuric acid; Reagent B: 165 mmolL^-1^ xylenol orange, 165 mmolL^-1^ sorbitol; A:B volume ratio 1:10), incubated in a 30 °C water bath for color development for 30 min, and the absorbance at 560 nm was measured. A standard curve for H_2_O_2_ was prepared using the same method, and the H_2_O_2_ content in peanut leaves was calculated.

### Superoxide anion generation rate (O_2_^∙-^) measurement

The generation rate of Superoxide Anion (O_2_^∙-^) was determined according to the method of Wang Aiguo et al. [43]. 1 g of peanut leaves from different treatments were ground into powder in liquid nitrogen, placed in a centrifuge tube with 4 mL of 50 mmolL^-1^ phosphate buffer (pH 7.8), and mixed well. The mixture was then centrifuged at 10,000 g at 4 °C for 20 min, and the supernatant was used as the test liquid for O_2_^∙-^ generation. 0.5 mL of test liquid was added to 0.5 mL of 50 mmolL^-1^ phosphate buffer (pH 7.8) and 1 mL of 1 mmolL^-1^ hydroxylamine hydrochloride, mixed well, and incubated at 25 °C for 1 h. Then, 2 mL each of 17 mmolL^-1^ p-aminobenzenesulfonic acid and 17 mmolL^-1^ α-naphthylamine were added, mixed well, and incubated at 25 °C for 20 min. The absorbance at 530 nm was measured. A NO_2_^-^ standard curve was prepared using the same method, and the generation rate of O_2_^∙-^ in peanut leaves was calculated.

### Antioxidant enzyme activity measurement

The activities of Peroxidase (POD), Catalase (CAT), Superoxide Dismutase (SOD), and Ascorbate Peroxidase (APX) were measured using commercial enzyme activity detection kits (Solarbio) according to the manufacturer’s instructions.

### Data processing

The data were analyzed statistically using SPSS Statistics 26 software. One-way ANOVA and Duncan’s multiple range test were utilized for significance testing, with a significance level set at *P* < 0.05.

## Results

### Induced resistance effects of exogenous calcium treatment against peanut web blotch

**Fig. 1:**
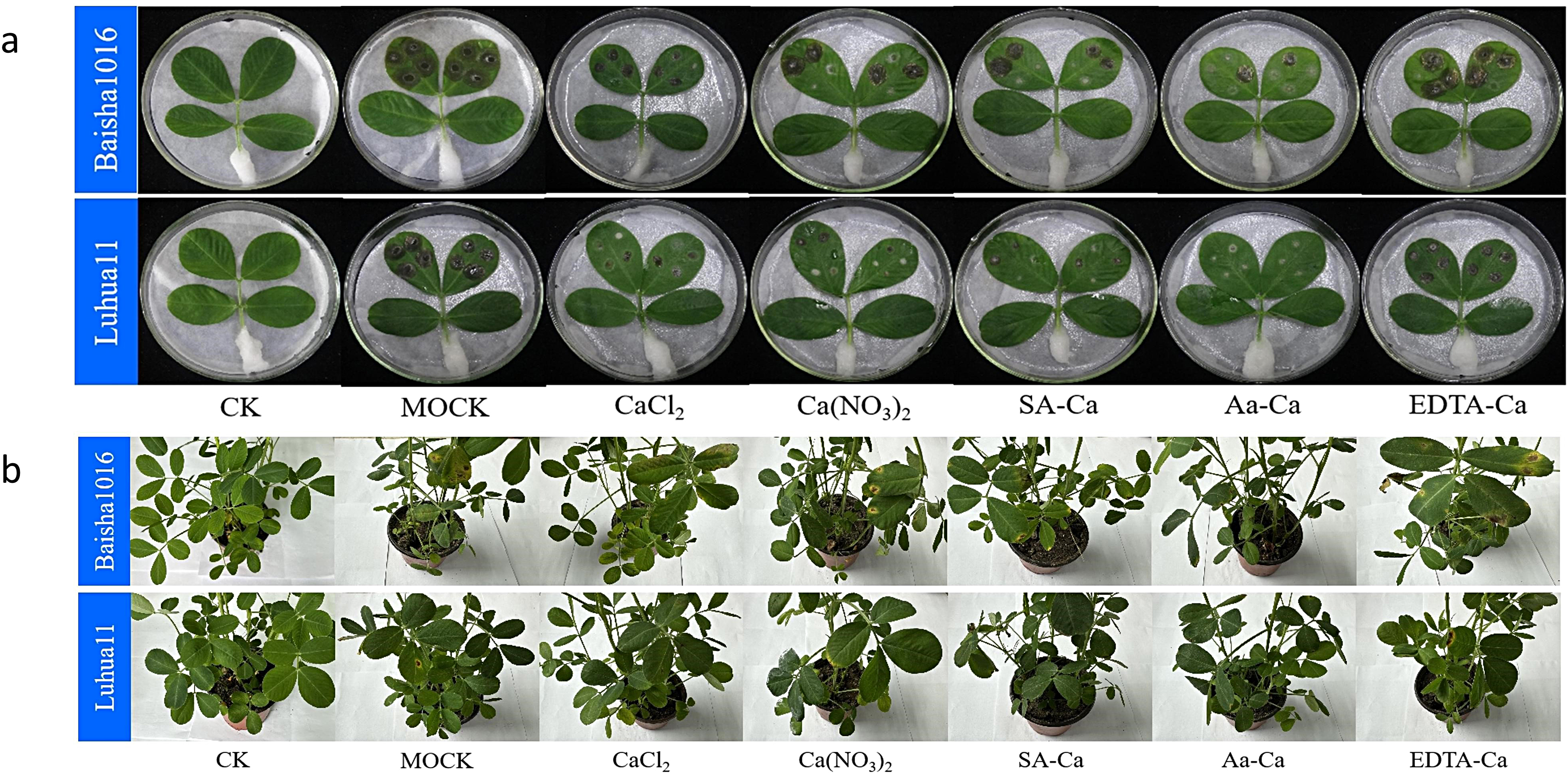
Exogenous calcium reduces the incidence of peanut leaf disease. **a** In-Vitro. **b** In-Vivo

**Fig. 2:**
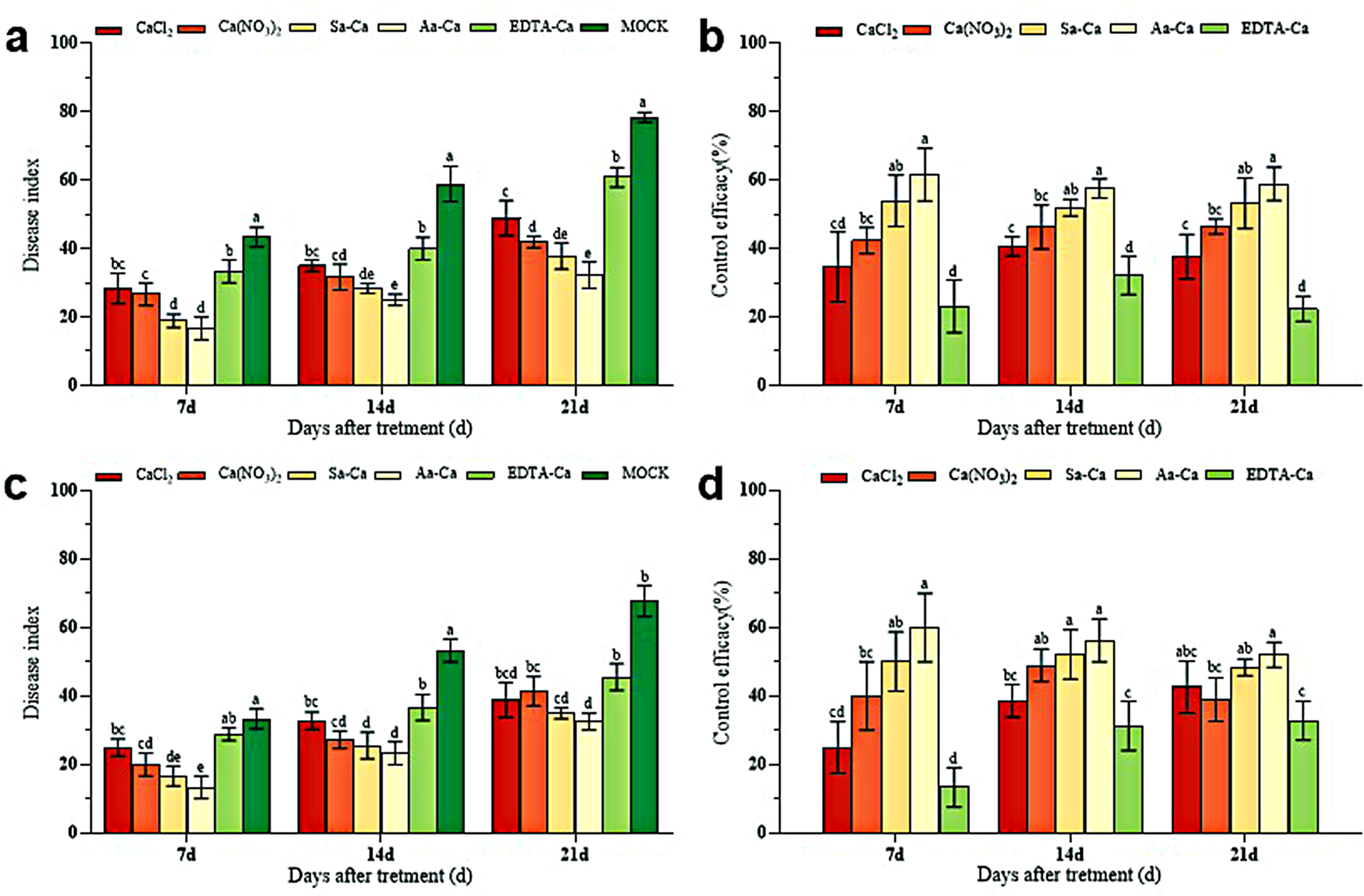
Effect of foliar application of exogenous calcium on inducing resistance in peanuts. **a** Baisha 1016 inoculated *Phoma arachidicola* Disease index. **b** Baisha 1016 inoculated *Phoma arachidicola* resistance-inducing effect. **c** Luhua 11 inoculated *Phoma arachidicola* Disease index. **d** Luhua 11 inoculated *Phoma arachidicola* resistance-inducing effect. *Note*: Error bars represented standard deviation (*n* = 3); Different letters indicated significant difference at 0.05 level by Duncan; The same as below

**Fig. 3:**
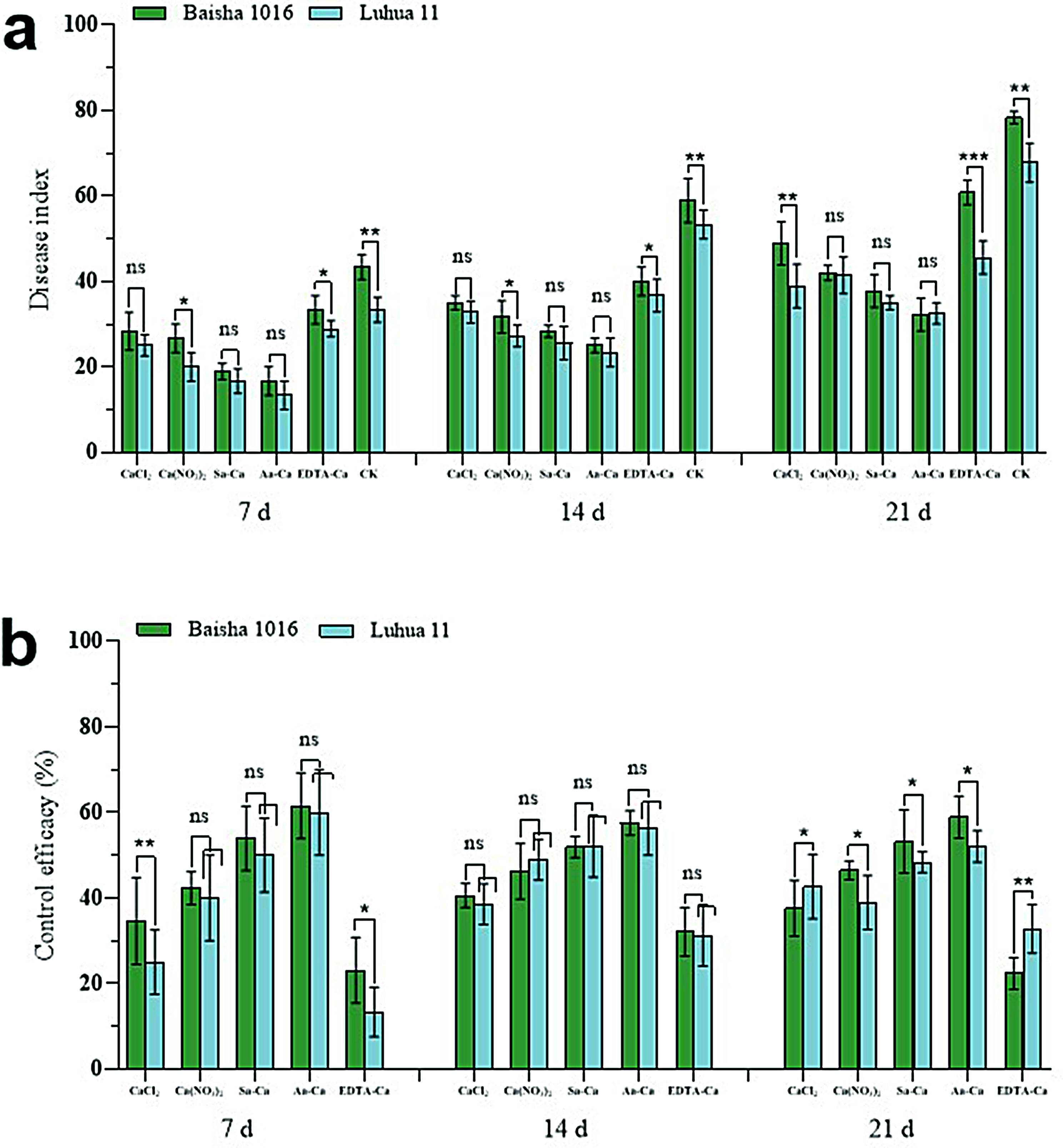
Comparison of induced resistance effects among different peanut varieties

Under greenhouse cultivation conditions, foliar application of five different exogenous calcium treatments showed a certain degree of induced resistance against peanut web blotch disease (Fig. 1). The disease index of peanut web blotch post-exogenous calcium treatment was lower compared to the control, with amino acid calcium and sorbitol calcium treatments resulting in the lowest disease index (Fig. 2). 7 days post-treatment, amino acid calcium showed the best induced resistance effect, reaching 61.54% (Baisha) and 60% (Luhua), followed by sorbitol calcium and calcium nitrate at 53.94% (Baisha) and 50% (Luhua), 42.31% (Baisha) and 39.99% (Luhua) respectively, and lastly calcium chloride and EDTA-Ca at 34.61% (Baisha) and 24.99% (Luhua), 23.07% (Baisha) and 13.32% (Luhua). 14 days post-treatment, the induced resistance effects of calcium chloride, calcium nitrate, sorbitol calcium, amino acid calcium, and EDTA-Ca on peanut web blotch disease were 40.57%, 46.23%, 51.89%, 57.55%, and 32.08% (Baisha), and 38.54%, 48.95%, 52.08%, 56.25%, and 31.25% (Luhua), respectively. 21 days post-treatment, the induced resistance effects of calcium chloride, calcium nitrate, sorbitol calcium, amino acid calcium, and EDTA-Ca on peanut web blotch disease were 37.59%, 46.45%, 53.19%, 58.86%, and 22.34% (Baisha), and 42.63%, 38.94%, 48.36%, 52.05%, and 32.79% (Luhua), respectively. Among these, the overall disease resistance effect of exogenous calcium on Luhua 11 was better than that on Baisha 1016 (Fig. 3).

### Effects of exogenous calcium treatments on the membrane permeability of peanut leaves

**Fig. 4:**
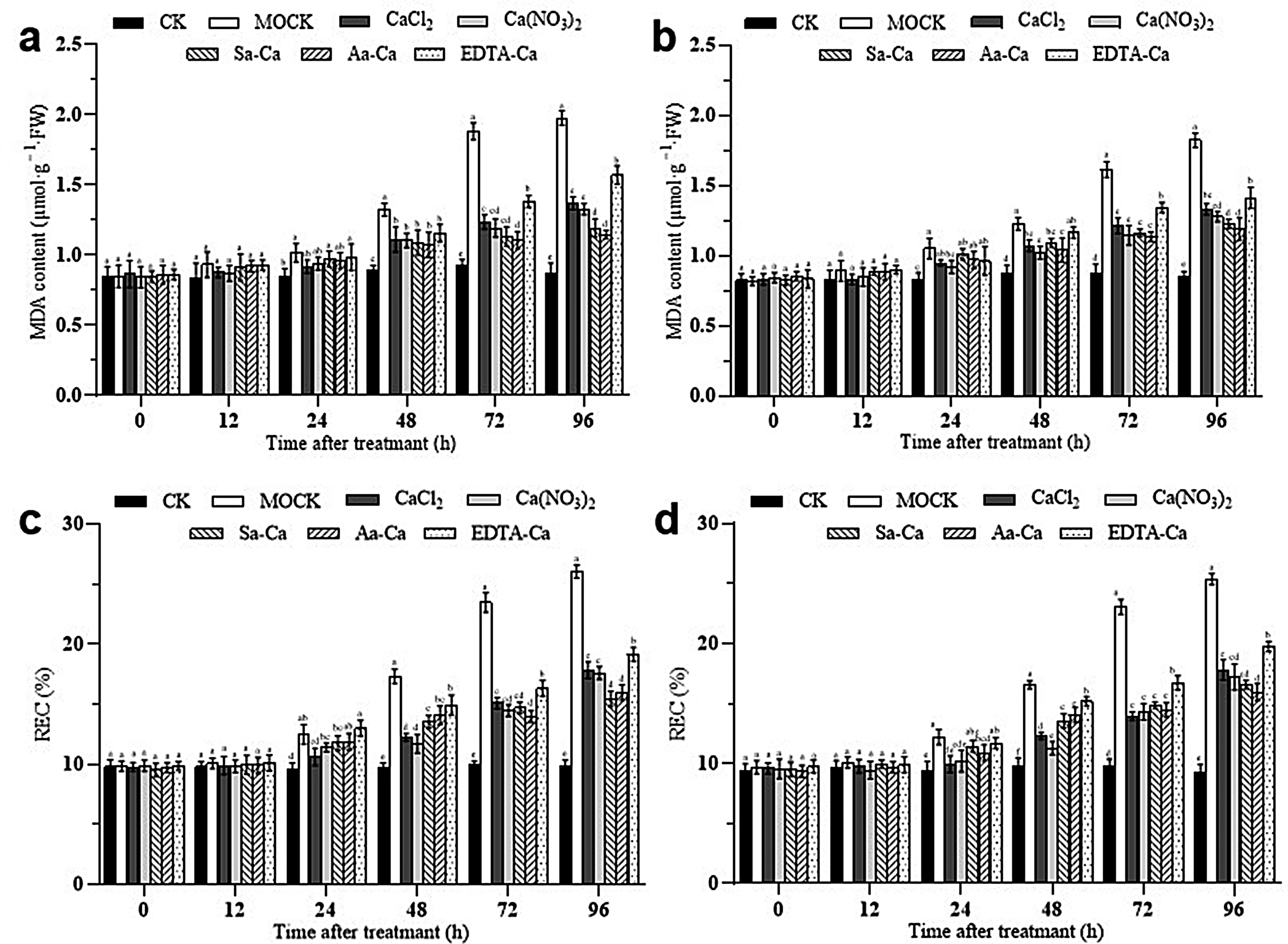
Effect of exogenous calcium treatments on the MDA content of peanut leaves. **a** Baisha 1016 inoculated *Phoma arachidicola.***b** Luhua 11 inoculated *Phoma arachidicol.* Effect of exogenous calcium treatments on the REC content of peanut leaves. **c** Baisha 1016 inoculated *Phoma arachidicola.***d** Luhua 11 inoculated *Phoma arachidicol*

MDA content in peanut leaves without inoculation remained unchanged over time, but gradually increased after inoculation with the web blotch pathogen. Compared to the leaves inoculated post-water spray, the MDA content in leaves treated with exogenous calcium and then inoculated was consistently lower. The most significant difference was observed at 72 h post-treatment (Fig. 4ab), where the MDA content in leaves treated with calcium chloride, calcium nitrate, sorbitol calcium, amino acid calcium, and EDTA-Ca decreased by 34.04%, 36.70%, 39.89%, 40.96%, 26.60% (Baisha) and 24.84%, 29.19%, 27.95%, 29.81%, 16.77% (Luhua), respectively.

Following inoculation with the web blotch pathogen, the REC of peanut leaves gradually increased over time. At 72 h post-treatment, compared to water spray followed by inoculation, foliar application of exogenous calcium before inoculation significantly lowered the REC, showcasing decreases of 35.72%, 38.36%, 37.34%, 40.49%, 30.52% (Baisha) and 39.50%, 37.89%, 35.50%, 37.20%, 27.52% (Luhua), respectively (Fig. 4cd).

### Impact of exogenous calcium treatment on hydrogen peroxide content and superoxide anion production rate in peanut leaves

**Fig. 5:**
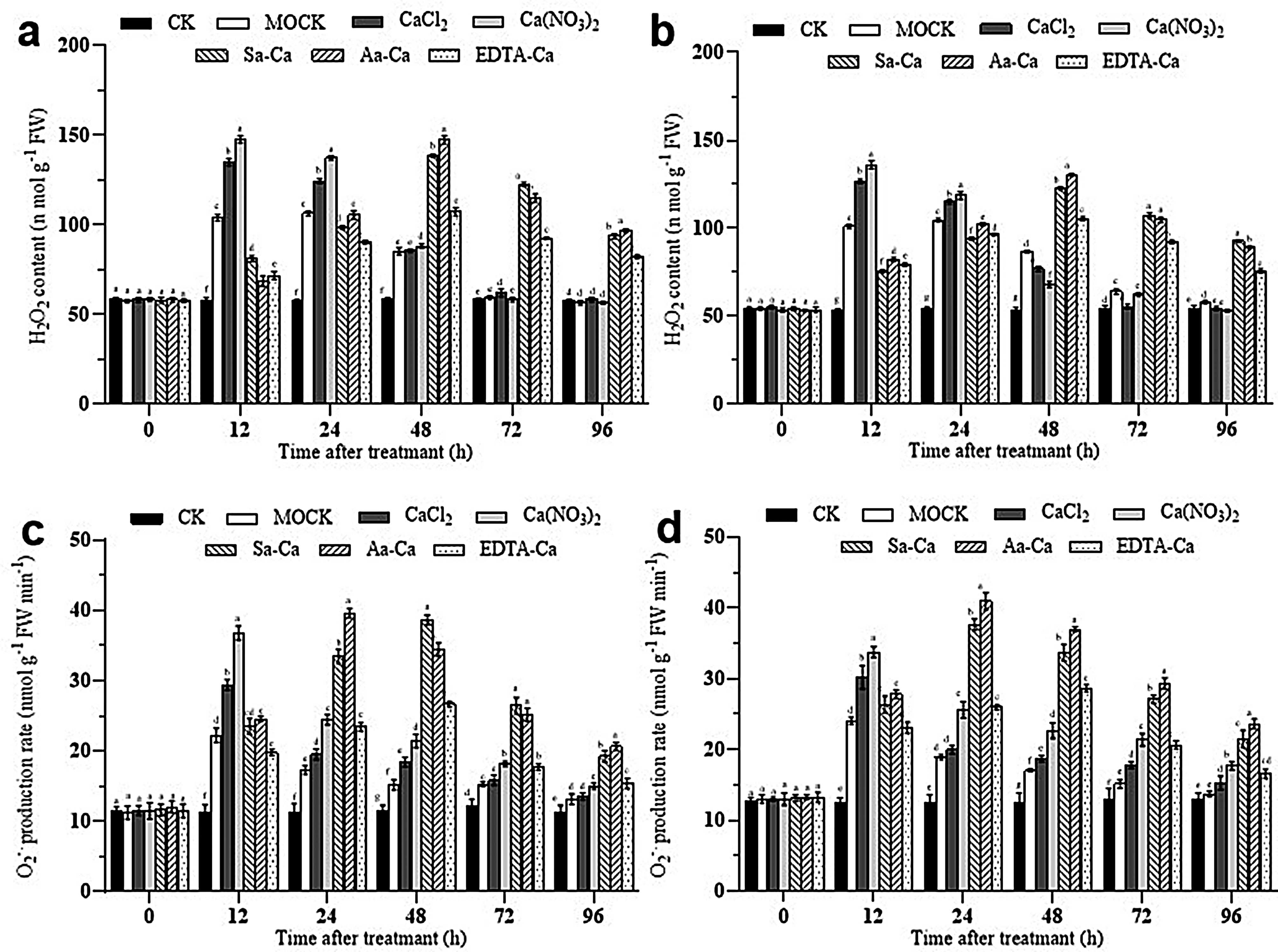
Effect of exogenous calcium treatments on the H_2_O_2_ content of peanut leaves. **a** Baisha 1016 inoculated *Phoma arachidicola.***b** Luhua 11 inoculated *Phoma arachidicol.* Effect of exogenous calcium treatments on the O_2_^∙-^ content of peanut leaves. **c** Baisha 1016 inoculated *Phoma arachidicola.***d** Luhua 11 inoculated *Phoma arachidicol*

Under calcium chloride and calcium nitrate treatments, the hydrogen peroxide (H_2_O_2_) content in peanut leaves rapidly increased after inoculation with the web blotch pathogen, peaking at 12 h, sharply decreasing after 24 h, and returning to baseline levels by 72 h. For sorbitol calcium, amino acid calcium, and EDTA-Ca treatments, the H_2_O_2_ content gradually increased over 0 to 48 h, peaked at 48 h, and then slowly decreased. This indicates that exogenous calcium treatments enhance the accumulation of H_2_O_2_ in peanut leaves, thus inducing an efficient and rapid response to infection by the web blotch pathogen (Fig. 5ab).

Following inoculation with the web blotch pathogen, all treatments influenced the rate of superoxide anion (O_2_^∙-^) production in peanut leaves to some extent. As shown in Fig. 4, foliar application of calcium chloride and calcium nitrate resulted in a rapid increase in the production rate of O_2_^∙-^ within 0 to 12 h, peaking at 12 h before quickly declining. For leaves treated with amino acid calcium and sorbitol calcium, the production rate of O_2_^∙-^ gradually rose over 0 to 24 h, peaking at 24 h, with amino acid calcium treatment leading to the fastest production rate. This demonstrates that exogenous calcium treatments can induce bursts of O_2_^∙-^ in peanut leaves, enhancing resistance against the web blotch pathogen (Fig. 5cd).

### Effects of exogenous calcium treatment on antioxidative enzyme activities in peanut leaves

**Fig. 6:**
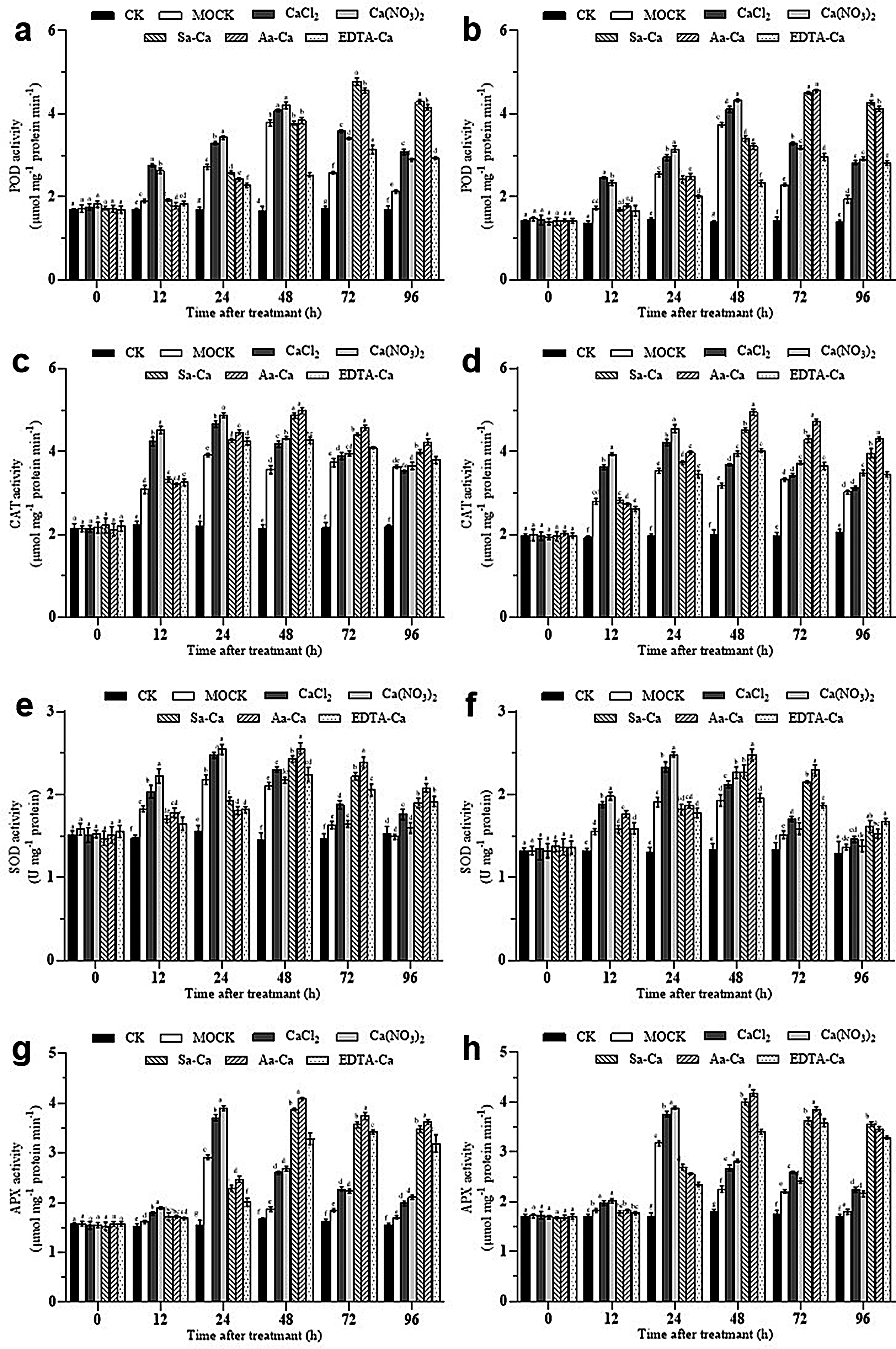
Effect of exogenous calcium treatments on the peroxidase system activities of peanut leaves. **a** POD Baisha 1016 inoculated *Phoma arachidicola.***b** POD Luhua 11 inoculated *Phoma arachidicol.***c** CAT Baisha 1016 inoculated *Phoma arachidicola.***d** CAT Luhua 11 inoculated *Phoma arachidicol.***e** SOD Baisha 1016 inoculated *Phoma arachidicola.***f** SOD Luhua 11 inoculated *Phoma arachidicol*. **g** APX Baisha 1016 inoculated *Phoma arachidicola.***h** APX Luhua 11 inoculated *Phoma arachidicol*

Following inoculation with the web blotch pathogen and exogenous calcium treatment, peroxidase (POD) activity in leaves of different peanut varieties was significantly enhanced (Fig. 6ab). In leaves inoculated post-water spray, POD activity rapidly rose within 0 to 48 h, peaking at 48 h before immediately dropping back to baseline; for leaves treated with calcium chloride and calcium nitrate and then inoculated, POD activity peaked at 24 h; leaves treated with sorbitol calcium and amino acid calcium and then inoculated saw a peak in POD activity at 48 h; with EDTA-Ca treatment, no distinct peak period was observed, with the highest POD activity recorded between 24 and 72 h.

Under exogenous calcium treatment, catalase (CAT) activity in peanut leaves was affected to varying degrees (Fig. 6cd). 24 h post-treatment, leaves treated with calcium chloride and calcium nitrate and then inoculated with the web blotch pathogen reached peak CAT activity levels, which then gradually declined; forty-eight hours post-treatment, leaves treated with sorbitol calcium and amino acid calcium and then inoculated reached peak CAT activity levels, which then gradually declined. Under EDTA-Ca treatment, CAT activity in peanut leaves continuously increased between 24 and 72 h without a clear peak.

Similarly, superoxide dismutase (SOD) activity was significantly enhanced in leaves of different peanut varieties following inoculation with the web blotch pathogen and exogenous calcium treatment (Fig. 6ef). In leaves inoculated post-water spray, SOD activity rapidly rose within 0 to 48 h, peaking at 48 h before quickly declining to baseline levels; leaves treated with calcium chloride and calcium nitrate and then inoculated reached peak SOD activity at 24 h; leaves treated with sorbitol calcium and amino acid calcium and then inoculated peaked in SOD activity at 48 h; with EDTA-Ca treatment, no distinct peak period was observed, with the highest SOD activity recorded between 24 and 72 h.

Following inoculation with the web blotch pathogen and exogenous calcium treatment, ascorbate peroxidase (APX) activity was significantly enhanced in leaves of different peanut varieties (Fig. 6gh). Leaves treated with calcium chloride and calcium nitrate and then inoculated experienced a sharp rise in APX activity between 12 and 24 h, peaking at 24 h before quickly declining, but remained consistently higher than APX activity in leaves inoculated post-water spray and untreated; leaves treated with sorbitol calcium and amino acid calcium and then inoculated reached peak APX activity at 48 h, which then slowly declined; under EDTA-Ca treatment, no distinct peak period was observed, with the highest APX activity recorded between 48 and 96 h.

## Discussion

The results of this experiment indicate that the application of five exogenous calcium treatments exhibited varying levels of induced resistance against peanut web blotch. Among these treatments, amino acid calcium was the most effective, inducing resistance in Baisha1016 and Luhua11 varieties by 61.54% and 60%, respectively. This was followed by sorbitol calcium and nitrate calcium, with induction effects of 53.94% and 50%, 42.31% and 39.99%, respectively, while calcium chloride and EDTA-Ca had the least effects, at 34.61% and 24.99%, 23.07% and 13.32%. Additionally, exogenous calcium treatments reduced the MDA content and REC in peanut leaves, enhanced the accumulation of H_2_O_2_ and O_2_^∙-^, and increased the activities of POD, CAT, SOD, and APX. This suggests that exogenous calcium treatments can induce peanut’s defense against web blotch through various pathways, including reducing cell membrane permeability, triggering ROS bursts, and activating antioxidant enzymes, thereby enhancing resistance to the disease.

Previous research results have also confirmed the role of calcium in inducing disease resistance. For instance, studies by Paula Júnior et al. have shown that spraying CaCl_2_ and CaSiO_3_ on bean leaves significantly reduces the disease index of bean plants infected with *Sclerotinia sclerotiorum* [44]; Yoon et al. found that treatments with different calcium compounds significantly reduce the susceptibility of peppers to *Botrytis cinerea* [45]. Thus, the use of calcium as an elicitor for controlling peanut diseases holds broad prospects.

The cell membrane is considered the first line of defense for plants against environmental stresses. When plants are subjected to various harmful stresses, the cell membrane gets damaged, significantly increasing its permeability. MDA content and REC are two main indicators of cell membrane permeability [46, 47]. Calcium treatments can stabilize plant epidermal cell membranes by reducing the leaf MDA content and REC, thereby mitigating pathogen invasion and proliferation. In contrast, a deficiency in calcium increases membrane permeability, allowing low molecular compounds (e.g., sugars, amino acids) to seep into the extraplastidial matrix of leaves and stem tissues, facilitating pathogen invasion and reproduction [48]. Exogenous calcium treatment enhances the stability of leaf epidermal cell membranes, activates the plant’s defense response to the web blotch pathogen, and effectively reduces the severity of peanut web blotch.

ROS are key defenders against pathogen invasion, either by directly killing pathogens or by slowing their entry into plant tissues. Under stress conditions, ROS such as H_2_O_2_ and O_2_^∙-^ are produced. These strong oxidizing agents can rapidly attack and cause damage to various biomolecules. Both H_2_O_2_ and O_2_^∙-^ can interact with polyunsaturated lipids in cell membranes, generating lipid peroxides that can lead to the destruction of biological membranes [49]. The application of exogenous calcium can induce an increase in H_2_O_2_ content and enhance the rate of O_2_^∙-^ production in peanut leaves. This indicates that under pathogen inoculation conditions, exogenous calcium-treated peanut leaves can rapidly accumulate more ROS, a process considered a key step in inducing further defense responses in plants. However, excessive ROS accumulation can lead to oxidative stress and ultimately cell death [49]. Plants have evolved many mechanisms to eliminate excess ROS throughout their evolutionary history, among which antioxidant enzymes are primary ROS scavengers, crucial for plant stress resistance [50, 51]. Exogenous calcium treatment significantly increased the activity of antioxidant enzymes, including POD, CAT, SOD, and APX, in peanut leaves, effectively eliminating excess ROS and maintaining ROS balance [50]. Overall, exogenous calcium treatment can induce peanut plant resistance to web blotch pathogen infection by regulating ROS metabolism to accumulate ROS and by activating antioxidant enzymes to eliminate excess ROS.

## Conclusions

In the context of sustainable agriculture, disease control strategies are increasingly considering the impact on the ecological environment and the protection of biodiversity among other issues. The use of exogenous inducers as an alternative to chemical fungicides for plant disease control presents a better option. Exogenous calcium as an inducer in the control of peanut diseases can reduce the impact of chemical fungicides on the environment, agricultural products, and human health. This experiment show that exogenous calcium has a certain inducing resistance effect on peanut web blotch. Exogenous calcium treatment can induce peanut to produce a defensive response through various pathways such as reducing the permeability of the cell membrane, triggering ROS bursts, and activating the activity of the antioxidant enzyme system, thereby improving the disease resistance of peanuts. This experiment provides a theoretical basis for controlling peanut diseases with exogenous calcium and offers reference significance for the prevention and control of diseases in other crops. However, the specific mechanisms of exogenous calcium treatment still require further research, including the analysis of regulatory networks of related genes and calcium signaling pathways. Moreover, in practical applications, it is necessary to further consider the optimal conditions and application methods for exogenous calcium treatments to maximize plant disease resistance.

## Data Availability

The author confirms that all data generated or analyzed during this study are included in this published article.
